# Role of Respiratory Pressure in Swallowing Function and Aspiration Risk in Parkinsonism

**DOI:** 10.1007/s00455-026-10939-2

**Published:** 2026-03-04

**Authors:** Minjung Kim, Seungmin Lee, Han-Joon Kim, Byung-Mo Oh, Han Gil Seo

**Affiliations:** 1 Department of Rehabilitation Medicine, Seoul National University Hospital, Seoul National University College of Medicine, 101 Daehak‑Ro, Jongno‑Gu, Seoul, 03080 Republic of Korea; 2 Department of Neurology, Seoul National University Hospital, Seoul National University College of Medicine, 101 Daehak‑Ro, Jongno‑Gu, Seoul, 03080 Republic of Korea; 3 Institute on Aging, Seoul National University College of Medicine, Seoul, Republic of Korea

**Keywords:** Parkinsonism, Dysphagia, Respiratory pressure, Aspiration, Respiratory muscle strength

## Abstract

Dysphagia is a prevalent and clinically significant non-motor symptom in Parkinson’s disease (PD) and atypical parkinsonism, leading to aspiration pneumonia and increased morbidity. Respiratory muscle weakness has been suggested as a contributor to impaired airway protection, but its clinical implications in parkinsonism remain underexplored. This retrospective study investigated the association between maximal respiratory pressure and dysphagia-related outcomes in individuals with PD and atypical parkinsonism. A total of 98 cases who underwent both videofluoroscopic swallowing study (VFSS) and respiratory pressure testing within three months were included. Maximal inspiratory pressure (MIP) and maximal expiratory pressure (MEP) were measured and normalized to age- and sex-matched predicted values. Swallowing function was evaluated using the Videofluoroscopic Dysphagia Scale (VDS), Penetration-Aspiration Scale (PAS), and Swallowing Disturbance Questionnaire (SDQ). Normalized MIP and MEP were significantly lower in the aspiration group (35.39 ± 22.62%, 40.69 ± 24.75%) than in the non-aspiration group (44.50 ± 21.79%, 50.30 ± 20.85%; *p* < 0.05, respectively). In atypical parkinsonism, normalized MIP and MEP were significantly lower in the aspiration group and was correlated with higher VDS scores (*r* = − 0.320, *r* = − 0.426; *p* < 0.05) and PAS scores (*r* = − 0.271, *r* = − 0.280; *p* < 0.05). Logistic regression showed that in atypical parkinsonism, each 1% decrease in normalized MEP was associated with a 4.7% increase in aspiration odds (adjusted OR = 0.953, 95% CI: 0.917–0.991). These findings suggest that reduced respiratory muscle strength may be linked to impaired swallowing function in parkinsonism, especially in atypical forms. Further prospective studies are needed to validate these results and clarify the role of respiratory muscle strength in dysphagia in parkinsonism.

**Supplementary Information:**

The online version contains supplementary material available at https://doi.org/10.1007/s00455-026-10939-2.

## Introduction

Parkinsonism refers to a clinical syndrome characterized by bradykinesia, rigidity, resting tremor, and postural instability [1]. When excluding secondary causes such as medications, toxins, or brain lesions, it is classified as primary parkinsonism, which includes Parkinson’s disease (PD) and atypical parkinsonism [2]. PD is one of the most prevalent neurodegenerative disorders worldwide, and its prevalence increases with age [3]. In South Korea, the prevalence of PD has steadily risen in parallel with the aging population [4]. According to data from the Health Insurance Review and Assessment Service, the number of patients with PD per 100,000 population increased from 41.4 in 2004 to 233.7 in 2022 [5]. The prevalence of atypical parkinsonism is less well documented in population-based studies. Both PD and atypical parkinsonism are associated with an increasing socioeconomic burden due to longer life expectancy, prolonged disease duration, and the need for long-term care [6, 7].

Dysphagia is one of the most common and serious non-motor symptoms associated with parkinsonism, significantly contributing to morbidity and mortality [8, 9]. A meta-analysis also estimated that over one-third of patients with PD experience dysphagia [10]. Atypical parkinsonism, including progressive supranuclear palsy (PSP), multiple system atrophy (MSA) and corticobasal degeneration (CBD), is less prevalent than idiopathic PD but is characterized by more rapid disease progression [2]. While the exact prevalence of dysphagia in atypical parkinsonism is not well established, recent data suggest that in PSP, dysphagia affects approximately 79% of patients, with estimates varying by assessment method [9]. Patients with atypical parkinsonism tend to develop dysphagia earlier and more severely, which increases the risk of respiratory complications such as aspiration pneumonia [9, 11]. Compared to the general population, patients with PD have an elevated risk of aspiration pneumonia, which is also associated with higher mortality [8]. Dysphagia can further lead to reduced oral intake, dehydration and malnutrition. These findings highlight the critical need for early detection and management of dysphagia in parkinsonism [12].

Expiratory muscle strength training (EMST) has been recommended as an evidence-based therapeutic intervention for dysphagia in PD, supported by multiple clinical trials and systematic reviews [13–15]. Previous studies have shown that EMST can improve swallowing safety and reduce penetration-aspiration scale (PAS) scores in patients with PD [14, 15]. EMST has also been explored as a potential intervention in atypical parkinsonism, with some studies reporting improvements in maximal expiratory pressure (MEP) [16], however, other is still in the feasibility stage [17]. Cough function has been identified as an important clinical predictor of dysphagia and aspiration in patients with dysphagia [18–20]. Effective cough requires a coordinated sequence of deep inspiration, glottic closure, and forceful expiration. These steps are physiologically dependent on both inspiratory and expiratory respiratory muscle strength, represented by maximal inspiratory pressure (MIP) and MEP [20–22]. Inadequate inspiratory strength can impair the ability to take a sufficient preparatory breath [23], while reduced expiratory strength compromises the generation of subglottic pressure necessary for expelling aspirated material [24, 25]. Thus, MIP and MEP may serve not only as indices of respiratory muscle function but also as potential contributors to airway protection through cough. Given that ineffective cough has been associated with increased aspiration risk and pneumonia in parkinsonism [26, 27], incorporating measures of MIP and MEP could provide clinically relevant information regarding swallowing safety and airway defense.

Therefore, the aim of this study is to investigate the association between maximal respiratory pressure and various dysphagia-related outcomes based on videofluoroscopic swallowing study (VFSS) findings in individuals with PD and atypical parkinsonism. Through this investigation, we seek to determine whether simple measurements of respiratory pressure could serve as practical indicators to guide further evaluation and monitoring of dysphagia.

## Method

### (1) Study Population

**Fig. 1:**
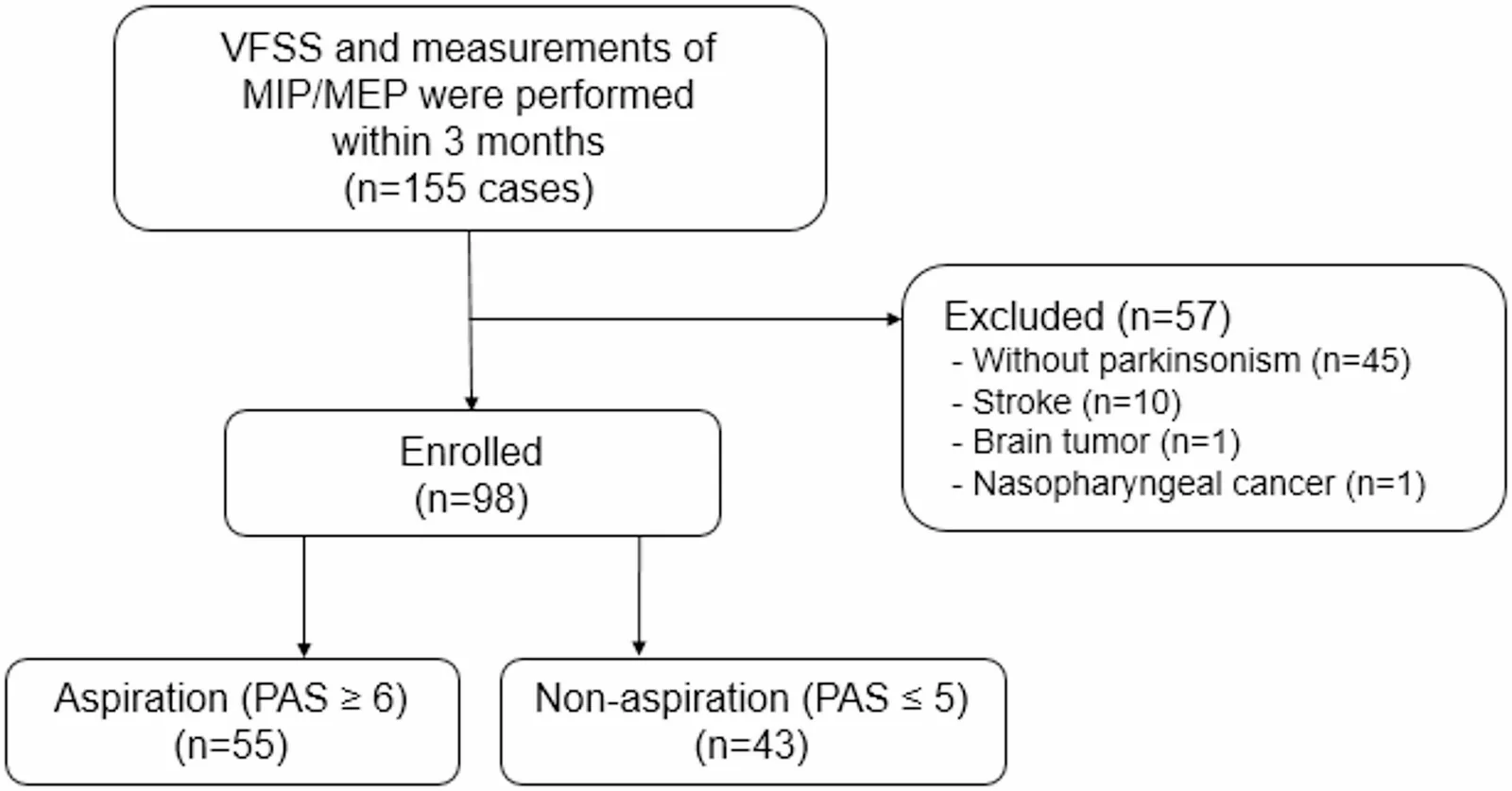
Flowchart of case enrollment. *VFSS* Videofluoroscopic swallowing study, *MIP* Maximal inspiratory pressure; MEP, Maximal expiratory pressure; PAS, Penetration-Aspiration scale

This study retrospectively reviewed cases of parkinsonism in which MIP and MEP were measured within three months of undergoing a VFSS at our outpatient clinic between January 2021 and December 2024. Data were extracted from electronic medical records using SUPREME 2.0, the Clinical Data Warehouse (CDW) platform of our hospital. Diagnoses of PD and PSP were made according to the MDS clinical diagnostic criteria [28, 29]; MSA was diagnosed based on the second consensus statement on the diagnosis of MSA [30]; CBD was diagnosed according to the Armstrong criteria [31], all by board-certified neurologists. Patients with comorbidities that could affect swallowing function, such as stroke, motor neuron disease, brain tumor, spinal cord injury, or nasopharyngeal cancer, were excluded. A total of 155 cases were initially identified, of which 57 met the exclusion criteria, resulting in 98 cases being included in the final analysis (Fig. 1). The study protocol was approved by the Institutional Review Board of our hospital (IRB No. 2504-043-1628).

### (2) Assessment

VFSS was conducted by two physicians using a modified version of Logemann’s protocol [32]. Patients were seated in a lateral position during the examination, and dynamic fluoroscopic imaging was obtained at 30 frames per second as they swallowed various consistencies of barium-contrasted materials, following standardized guidelines. Thin liquid was administered sequentially in volumes of 2 ml and 5 ml, followed by 30 ml if tolerated. Nectar-thick and honey-thick liquids were each provided in 5 ml portions, and solid consistency was offered in a small bite-sized amount. All test volumes were administered twice, except for the 30 ml thin liquid, which was administered once if tolerated. All VFSS images were recorded and later reviewed frame-by-frame by experienced clinicians to ensure consistent and accurate scoring.

Swallowing function was assessed over multiple trials, and penetration-aspiration events were evaluated using the PAS [33]. The Videofluoroscopic Dysphagia Scale (VDS) was also assessed based on VFSS findings. The VDS is a validated tool that quantifies swallowing impairment by scoring various functional components [34–36]. It consists of 14 items with a total score of 100, including abnormalities in the oral and pharyngeal phases, residue, and aspiration risk. This standardized approach allowed for a comprehensive assessment of dysphagia severity and aspiration risk in the study population.

MIP and MEP were measured using a respiratory pressure meter (MicroRPM, MD Spiro, Maine, USA). Participants were instructed to perform maximal inspiratory and expiratory efforts following a full exhalation or inhalation, respectively. During this task, participants were instructed to “blow out or inhale as hard as you can” to ensure maximal effort [37, 38]. MIP and MEP were measured three times, and the highest value was recorded [38]. A nose clip was used in all measurements to prevent nasal air leakage. These measured values were compared to normative data from healthy Korean adults matched for age and sex and expressed as a percentage [39]. These adjusted values were referred to as normalized MIP and normalized MEP (%).

The Swallowing Disturbance Questionnaire (SDQ) is a patient-reported screening tool for dysphagia in PD [40, 41]. In this study, the Korean version of the SDQ, consisting of 15 items, was obtained from the patients’ medical records at the time of VFSS [42]. All clinical assessments were conducted during the “on” state for patients taking antiparkinsonian medications.

### (3) Statistical Analysis

Descriptive statistics were analyzed using frequency analysis, and independent t-tests were used to compare group differences, presented as mean ± standard deviation. The differences between aspiration and non-aspiration groups across the modified Hoehn and Yahr (H&Y) stages and diagnostic subtypes were evaluated using chi-square tests. When expected cell counts were less than 5, Fisher’s exact test was applied. Additionally, independent t-tests were performed to compare raw MIP and MEP values between aspiration and non-aspiration groups. Pearson or Spearman correlation analyses were performed based on data distribution to examine the relationships between variables. Logistic regression analysis was used to identify factors associated with clinical outcomes. Both unadjusted and adjusted analyses were conducted, with odds ratios (ORs) and 95% confidence intervals (CIs) calculated. The adjusted model accounted for potential confounders related to aspiration or parkinsonism, including age, sex and H&Y stage. For the atypical parkinsonism group, diagnostic subtype was additionally included as a covariate. All statistical analyses were conducted using the Statistical Package for Social Sciences (version 25.0; SPSS Inc., Chicago, IL), and a P-value of less than 0.05 was considered statistically significant.

## Result

A total of 155 cases underwent VFSS for dysphagia, and MIP and MEP were measured within three months. After applying exclusion criteria, 98 cases were included in the final analysis. The mean duration between VFSS and MIP/MEP assessment was 10.93 ± 18.18 days. Among these, 55 cases with subglottic aspiration (PAS ≥ 6) were classified as the aspiration group, and 43 cases without material aspiration below the vocal folds (PAS ≤ 5) were classified as the non-aspiration group based on VFSS findings (Fig. 1). The general characteristics of the study population are summarized in Table 1 and Supplementary Table 1. Age, H&Y stage, disease duration, height, parkinsonism diagnosis and levodopa equivalent dose (LED) were compared between the two groups. With the exception of LED, there were no statistically significant differences between two groups in terms of age, sex, H&Y stage, or disease duration.

**Table 1:** Demographics and clinical characteristics of non-aspiration and aspiration groups

Variable	Overall (*n* = 98)	Non-aspiration (*n* = 43)	Aspiration (*n* = 55)	*P*
Age, yr	71.76 ± 9.33	70.00 ± 10.00	73.13 ± 8.62	0.107
Sex, n (M/F)	76/22	31/12	45/10	0.252
Height, cm	164.12 ± 7.91	162.70 ± 8.34	165.40 ± 7.36	0.135
Disease duration since onset, yr	6.36 ± 4.15	7.02 ± 4.75	5.84 ± 3.57	0.176
*Diagnosis*,* n*				0.916
PD	38	17	21	
MSA	41	19	22	
PSP	16	6	10	
CBD	3	1	2	
*H&Y stage (overall*^*a*^*)*,* n*				0.202
1–1.5	3	0	3	
2–2.5	14	6	8	
3	16	9	7	
4	38	16	22	
5	27	12	15	
*LED*				
Overall	796.49 ± 555.08	947.12 ± 601.98	677.12 ± 488.27	0.018*
PD	719.69 ± 330.26	719.31 ± 327.33	720.00 ± 341.09	0.995
Atypical parkinsonism	843.35 ± 653.94	1087.31 ± 690.19	651.14 ± 562.57	0.010*

All values are presented as mean ± standard deviation

*H&Y* Modified Hoehn and Yahr, *PD* Parkinson’s disease, *MSA* Multiple system atrophy, *PSP* Progressive supranuclear palsy, *CBD* Corticobasal degeneration, *LED* Levodopa equivalent dose

^a^ Detailed classification of diagnostic subtypes is provided in Supplementary Table 1

* P value < 0.05

Table 2 presents the differences in swallowing and respiratory assessments between the aspiration and non-aspiration groups. The non-aspiration group demonstrated a mean MIP of 44.50 ± 21.79% and a mean MEP of 50.30 ± 20.85% of the predicted values, whereas the aspiration group showed a mean MIP of 35.39 ± 22.62% and a mean MEP of 40.69 ± 24.75%. When comparing the two groups regardless of diagnostic subtype, the aspiration group had significantly higher VDS scores (*p* < 0.001) and lower normalized MEP values (*p* = 0.039). The difference in VDS scores between the two groups remained statistically significant in subgroup analyses of both PD (*p* < 0.001) and atypical parkinsonism (*p* = 0.002). However, while normalized MEP did not significantly differ between aspiration and non-aspiration groups in PD (*p* = 0.537), a significant difference was observed in atypical parkinsonism (*p* = 0.034).

**Table 2:** Difference in swallowing and respiratory assessments between non-aspiration and aspiration group

Variable	Non-aspiration	Aspiration	*P*
Overall	*n* = 43	*n* = 55	
VDS	28.94 ± 15.59	46.44 ± 14.18	< 0.001*
SDQ	14.25 ± 6.69	14.88 ± 8.66	0.715
MIP, cmH₂O	35.63 ± 17.85	29.96 ± 19.97	0.142
MEP, cmH₂O	51.19 ± 24.97	43.07 ± 25.07	0.115
Normalized MIP, %	44.50 ± 21.79	35.39 ± 22.62	0.046*
Normalized MEP, %	50.30 ± 20.85	40.69 ± 24.75	0.039*

PD	*n* = 17	*n* = 21	
VDS	20.76 ± 13.11	46.24 ± 14.53	< 0.001*
SDQ	11.84 ± 6.97	15.43 ± 9.17	0.223
MIP, cmH₂O	38.00 ± 19.31	30.86 ± 16.30	0.234
MEP, cmH₂O	53.35 ± 20.32	49.67 ± 23.68	0.609
Normalized MIP, %	48.69 ± 22.73	37.44 ± 19.00	0.113
Normalized MEP, %	55.10 ± 20.32	50.69 ± 27.47	0.537

Atypical Parkinsonism	*n* = 26	*n* = 34	
VDS	34.29 ± 14.94	46.56 ± 14.17	0.002*
SDQ	16.00 ± 6.05	14.62 ± 8.55	0.516
MIP, cmH₂O	34.08 ± 17.03	29.41 ± 22.16	0.360
MEP, cmH₂O	49.77 ± 27.89	39.00 ± 25.38	0.130
Normalized MIP, %	41.77 ± 21.16	34.13 ± 24.78	0.102
Normalized MEP, %	47.17 ± 23.20	34.52 ± 21.03	0.034*

All values are presented as mean ± standard deviation

*VDS* Videofluoroscopic dysphagia scale, *SDQ* Swallowing disturbance questionnaire, *MIP* Maximal inspiratory pressure, *MEP* Maximal expiratory pressure, *PD* Parkinson’s disease

* P value < 0.05

Table 3 summarizes the correlations between respiratory pressures and swallowing function. Overall, lower normalized MIP (*r* = − 0.309, *p* = 0.002) and MEP (*r* = − 0.409, *p* < 0.001) were significantly associated with higher total VDS scores. Regarding the VDS subscores, the oral phase subscore was correlated with both normalized MIP (*r* = − 0.463, *p* < 0.001) and MEP (*r* = − 0.506, *p* < 0.001). In subgroup analysis, patients with PD showed a significant association between the oral phase subscore and normalized MEP (*r* = − 0.345, *p* = 0.034), but no significant correlation with the total VDS score. In atypical parkinsonism, lower normalized MIP (*r* = − 0.320, *p* = 0.013) and MEP (*r* = − 0.426, *p* = 0.001) were significantly associated with higher total VDS scores. In this group, the oral phase subscore was significantly associated with both normalized MIP (*r* = − 0.583, *p* < 0.001) and MEP (*r* = − 0.565, *p* < 0.001), while the pharyngeal phase subscore was associated only with MEP (*r* = − 0.293, *p* = 0.023). No significant correlation was observed between SDQ scores and normalized MIP or MEP overall. Normalized MIP showed a significant correlation with PAS scores in the overall participants (*r* = − 0.315, *p* = 0.002), including those with PD and atypical parkinsonism. In atypical parkinsonism, lower normalized MEP (*r* = − 0.280, *p* = 0.031) was similarly associated with higher PAS scores.

**Table 3:** Correlation between respiratory pressure and swallowing functions

Variable	Overall		PD		Atypical parkinsonism	
	Coefficient	*P*	Coefficient	*P*	Coefficient	*P*
*Normalized MIP*,* %*						
VDS	−0.309	0.002*	−0.290	0.077	−0.320	0.013*
Oral phase	−0.463	< 0.001*	−0.213	0.199	−0.583	< 0.001*
Pharyngeal phase	−0.165	0.105	−0.274	0.096	−0.228	0.079
SDQ	−0.163	0.133	−0.136	0.466	−0.169	0.217
PAS	−0.315	0.002*	−0.395	0.014*	−0.271	0.036*
*Normalized MEP*,* %*						
VDS	−0.409	< 0.001*	−0.266	0.106	−0.426	0.001*
Oral phase	−0.506	< 0.001*	−0.345	0.034*	−0.565	< 0.001*
Pharyngeal phase	−0.270	0.007*	−0.182	0.273	−0.293	0.023*
SDQ	−0.171	0.115	−0.119	0.525	−0.121	0.380
PAS	−0.272	0.007*	−0.271	0.100	−0.280	0.031*

*VDS* Videofluoroscopic dysphagia scale, *SDQ* Swallowing disturbance questionnaire, *PAS* Penetration-Aspiration scale, *MIP* Maximal inspiratory pressure, *MEP* Maximal expiratory pressure, *PD* Parkinson’s disease

* P value < 0.05

Table 4 displays the association between aspiration and respiratory pressure. Logistic regression analysis revealed that, in atypical parkinsonism, lower normalized MEP was significantly associated with an increased risk of aspiration. In the adjusted model, the odds ratio was 0.953 (95% CI: 0.917–0.991), indicating that each 1% decrease in normalized MEP increased the odds of aspiration by approximately 4.7%. These findings suggest that even small reductions in expiratory pressure may contribute to elevated aspiration risk.

**Table 4:** Association between aspiration and respiratory pressure

Variable	Unadjusted analysis		Adjusted analysis^a^	
	OR (95% CI)	*P*	OR (95% CI)	*P*
*Overall*				
Normalized MIP, %	0.982 (0.963–1.000)	0.051	0.977 (0.956–0.999)	0.037*
Normalized MEP, %	0.982 (0.964–0.999)	0.048*	0.975 (0.953–0.997)	0.028*
*PD*				
Normalized MIP, %	0.973 (0.940–1.007)	0.116	0.968 (0.930–1.008)	0.112
Normalized MEP, %	0.991 (0.963–1.020)	0.549	0.991 (0.960–1.024)	0.595
*Atypical parkinsonism*				
Normalized MIP, %	0.986 (0.964–1.008)	0.211	0.985 (0.959–1.012)	0.267
Normalized MEP, %	0.974 (0.950–0.998)	0.037*	0.953 (0.917–0.991)	0.015*

*OR* Odds ratio, *CI* Confidence interval, *MIP* Maximal inspiratory pressure, *MEP* Maximal expiratory pressure, *PD* Parkinson’s disease

^a^ Adjusted for age, sex, Hoehn and Yahr stage, and diagnostic subtype

* P value < 0.05

## Discussion

To the best of our knowledge, this study is the first to demonstrate that respiratory muscle strength is significantly associated with the severity of dysphagia and the risk of aspiration in patients with parkinsonism. Both MIP and MEP were associated with the VDS in patients with parkinsonism, especially in atypical parkinsonism. Although lower MIP showed a significant correlation with PAS scores in correlation analysis, this association was not supported in group comparisons or logistic regression. Lower MEP demonstrated consistent and significant associations with swallowing dysfunction across group comparisons, correlation, and regression analyses. These associations were further confirmed in subgroup analyses, particularly within atypical parkinsonism. As maximal respiratory pressure reflects respiratory muscle strength, these findings suggest that expiratory muscle weakness, may play an important role in the pathophysiology of aspiration.

The association between respiratory muscle strength and swallowing function observed in this study may be attributable to a direct pathophysiological link, although it could also partly reflect a more general decline in systemic function related to disease progression. Swallowing and respiration are closely coordinated processes, sharing anatomical structures such as the pharynx and larynx and being organically regulated by central pattern generators in the brainstem [43, 44]. During a normal swallow, a brief cessation of breathing known as ‘swallowing apnea’ occurs, which is thought to play an essential role in airway protection [45]. Although expiratory muscle strength is traditionally emphasized for its role in airway clearance via cough, prior studies suggest that it may also contribute to airway protection by generating sufficient subglottic air pressure [46]. Adequate subglottic pressure is considered important for expelling material that has penetrated the airway back into the pharynx or clearing aspirated material [21]. In this context, the decreased MEP observed in our study, reflecting expiratory muscle weakness, may compromise the ability to generate adequate subglottic pressure, thereby increasing vulnerability to aspiration. While direct evidence linking MEP to swallowing outcomes in parkinsonism is still limited, this possible mechanistic relationship provides a rationale for further investigation and may help explain why interventions such as EMST have been reported to improve swallowing function in parkinsonism [15].

Previous studies in stroke patients have reported associations between respiratory muscle strength and dysphagia severity, particularly emphasizing the role of MEP in airway protection via effective coughing [47, 48]. Many studies on dysphagia in PD have demonstrated the potential benefits of EMST, and it has been increasingly recognized in the literature as an evidence-based therapeutic approach [13, 14, 49]. However, research on the relationship between expiratory muscle strength and dysphagia in PD remains limited, and there is a lack of studies addressing specific interventions, such as EMST, in atypical parkinsonism. In our study, the strength of association between respiratory muscle weakness and aspiration differed between PD and atypical parkinsonism. The absence of a significant association in the PD group may be attributable to differences in disease progression, compensatory mechanisms, or relatively preserved bulbar function during early stages of PD, in contrast to atypical parkinsonism [10, 11, 50, 51].

The observed differences between PD and atypical parkinsonism in the relationship between expiratory muscle strength and aspiration may reflect distinct pathophysiological mechanisms underlying dysphagia and respiratory dysfunction. Dysphagia in PD is relatively well studied and, in its early stages, is primarily characterized by oral phase involvement due to movement disorders associated with dopamine depletion and basal ganglia dysfunction [50, 52, 53]. In contrast, atypical parkinsonism tends to exhibit more rapid disease progression and earlier brainstem involvement, resulting in more severe bulbar dysfunction and earlier onset of dysphagia [11, 54]. In this study, both MIP and MEP were markedly reduced to approximately half of the predicted values across all patients with parkinsonism. However, respiratory dysfunction appeared to manifest differently between PD and atypical parkinsonism. Although reduction in MIP and MEP are known to occur in people with PD [55], existing but limited research suggests that respiratory involvement may be more pronounced in atypical parkinsonism, particularly in MSA [56]. This may explain the more statistically significant association between expiratory muscle weakness and aspiration in atypical parkinsonism, possibly reflecting broader involvement of central and peripheral motor pathways that influence cough effectiveness and subglottic pressure generation [21, 57].

In addition to pathophysiological differences, variations in responsiveness to levodopa may also contribute to the differences observed between PD and atypical parkinsonism. In PD, although levodopa has been shown to improve swallowing efficiency, it has minimal effect on swallowing safety or aspiration prevention [53]. Some studies have indicated that levodopa can enhance respiratory function by acting on respiratory muscles [58]. In this study, all patients were evaluated during the “on” state while taking levodopa. In patients with PD, it is possible that respiratory function may be partially enhanced during the “on” state, while swallowing safety might be less responsive to dopaminergic medication. This difference in responsiveness could have attenuated the observed association between respiratory function and dysphagia in PD. In contrast, atypical parkinsonism is generally less responsive to levodopa [59], which may partially explain the relatively stronger correlations observed in this group. These findings raise the possibility that different results could be obtained if PD patients were assessed during the “off” state.

​Our findings indicate that while MEP may be associated with aspiration risk, the strength of this association was relatively weak. In atypical parkinsonism, MEP showed correlations with dysphagia severity indices, such as the VDS score and the PAS, suggesting a possible relationship between expiratory muscle strength and swallowing function in this population. While further research is needed to determine the clinical utility of MEP, these findings support its potential relevance as a complementary measure to VFSS in the monitoring or triaging of dysphagia, particularly in patients with parkinsonism.

Our study included patients with relatively advanced parkinsonism. Many participants showed reduction in respiratory pressure compared to age-matched value and severe swallowing impairment, which may have influenced the strength of the associations observed. While this limits the generalizability of our findings to milder populations, it also provides valuable insights into patients at higher risk for aspiration. For clinicians, these findings may underscore the need to carefully monitor respiratory muscle strength in advanced parkinsonism [12, 21], where swallowing impairment is more prevalent and management of aspiration risk becomes particularly important.

There are several limitations to this study. First, this was a retrospective study conducted at a single tertiary care center, and the relatively small sample size, especially within diagnostic subgroups, may limit the generalizability of the findings. Additionally, due to the retrospective design, data on potential confounding variables that could influence results may have been unavailable. Disease-specific severity scales such as the progressive supranuclear palsy rating scale [60] or Unified Multiple System Atrophy Rating Scale [61] were not routinely available in the clinical records, limiting our ability to accurately characterize disease stage across atypical parkinsonism. Second, the cross-sectional nature of the study precludes establishing causal relationships between respiratory pressure and swallowing function. Although normalized values were adjusted for age and sex, other confounding factors such as nutritional status or sarcopenia were not considered. Additionally, although the average interval between VFSS and MIP/MEP assessment was approximately 10 days, the data were not collected on the same day, and this temporal discrepancy may have introduced variability due to changes in clinical status between assessments. Moreover, the VFSS protocol did not include larger boluses, more than 30 ml. Given that airway invasion in parkinsonism is often more apparent with large volume, our protocol may not have fully captured aspiration events in participants with airway protective deficits. This should be considered when interpreting our findings. Furthermore, as this was an exploratory analysis, statistical correction for multiple comparisons was not applied. As both normalized MIP and MEP were evaluated as predictors, the potential for type I error should be considered when interpreting the results. Future research should include prospective and longitudinal studies examining the relationship between respiratory function and dysphagia progression across different types of parkinsonism. In addition, interventional trials evaluating the effect of EMST on aspiration risk and swallowing outcomes would be of significant clinical value, especially in atypical parkinsonism where current treatment options are limited.

## Conclusion

This study suggests that respiratory pressure may be associated with swallowing function and aspiration risk in patients with parkinsonism, particularly in those with atypical parkinsonism. These findings highlight the potential clinical utility of respiratory pressure measurements as a simple tool for monitoring dysphagia progression. While the overall associations were modest, the consistent relationship between reduced MEP and aspiration-related outcomes in atypical parkinsonism suggests that expiratory muscle weakness may play an important role in swallowing safety. Further prospective studies are needed to confirm these findings and clarify the association between respiratory function and dysphagia in parkinsonism.

## Electronic Supplementary Material

Below is the link to the electronic supplementary material.

Supplementary Material 1

## Data Availability

The data that support the findings of this study are available from the corresponding author upon reasonable request.

## Declarations

**Consent** This study did not require informed consent because it was a retrospective study with ethical approval.
