# Supplementary material for: Role of Respiratory Pressure in Swallowing Function and Aspiration Risk in Parkinsonism
Source: Dysphagia. 2026 Mar 4;41(5):900–9. doi: 10.1007/s00455-026-10939-2 (PMC13683480; doi:10.1007/s00455-026-10939-2)
Supplement: Supplementary file 1 [file 455_2026_10939_MOESM1_ESM.docx]

**Supplementary Table 1** Demographics and clinical characteristics of non-aspiration and aspiration groups

| **Variable** | **Overall**  **(n=98)** | **Non-aspiration (n=43)** | **Aspiration  (n=55)** |
| --- | --- | --- | --- |
| H&Y stage (PD), n |  |  |  |
| 1–1.5 | 3 | 0 | 3 |
| 2–2.5 | 10 | 5 | 5 |
| 3 | 8 | 4 | 4 |
| 4 | 14 | 6 | 8 |
| 5 | 3 | 2 | 1 |
| H&Y stage (MSA), n |  |  |  |
| 1–1.5 | 0 | 0 | 0 |
| 2–2.5 | 0 | 0 | 0 |
| 3 | 5 | 3 | 2 |
| 4 | 14 | 7 | 7 |
| 5 | 22 | 9 | 13 |
| H&Y stage (PSP), n |  |  |  |
| 1–1.5 | 0 | 0 | 0 |
| 2–2.5 | 2 | 0 | 2 |
| 3 | 2 | 2 | 0 |
| 4 | 10 | 3 | 7 |
| 5 | 2 | 1 | 1 |
| H&Y stage (CBD), n |  |  |  |
| 1–1.5 | 0 | 0 | 0 |
| 2–2.5 | 2 | 1 | 1 |
| 3 | 1 | **0** | **1** |
| 4 | 0 | 0 | 0 |
| 5 | 0 | 0 | 0 |

All values are presented as mean ± standard deviation

*H&Y* Modified Hoehn and Yahr, *PD* Parkinson’s disease, *MSA* Multiples system atrophy, *PSP* Progressive supranuclear palsy, *CBD* Corticobasal degeneration

* P value <0.05
